# Hybrid dielectric–graphene nanostructured SERS substrates for antibody sensing

**DOI:** 10.1039/d5na00847f

**Published:** 2025-11-18

**Authors:** Javier Redolat, Miguel Sinusia Lozano, María Camarena Pérez, Ignacio González-Llácer, Sofiya Zorina, Eva Zafra, Mar Alonso Chornet, Evelyn Díaz-Escobar, Víctor J. Gómez, Alejandro Martínez, Elena Pinilla-Cienfuegos

**Affiliations:** a Nanophotonics Technology Center (NTC), Universitat Politècnica de València Valencia Spain epinilla@ntc.upv.es +34 963 87 97 36; b Department of Microelectronics, Faculty of Electrical Engineering, Mathematics and Computer Science, Delft University of Technology Delft The Netherlands; c École des Mines de Saint-Étienne Saint-Étienne France

## Abstract

We present a hybrid surface-enhanced Raman spectroscopy (SERS) platform based on a nanostructured silicon substrate integrated with functionalized graphene for the selective detection of biomolecules such as prolactin and SARS-CoV-2 antibodies. The high-index substrate comprises an array of subwavelength silicon nanopillars that support Mie-type optical resonances, enabling strong electromagnetic field confinement with minimal heating and optical losses. Graphene monolayers are transferred onto the nanopillar array and functionalized using 1-pyrenebutanoic acid succinimidyl ester (PBASE), thus facilitating the selective immobilization of target antibodies *via* π–π interactions and covalent bonding. Graphene transfer, functionalization, and analyte binding are confirmed by the SERS enhancement, which enables label-free detection at low laser power, avoiding photodamage and ensuring compatibility with sensitive biomolecules. Strain and doping analysis, performed through Raman vector decomposition, reveals distinct responses associated with each antibody, validating the sensor's capability for molecular discrimination.

## Abstract 2

Hybrid dielectric–graphene nanostructured substrates enable highly sensitive, low-power SERS detection of antibodies.

## Graphical abstract

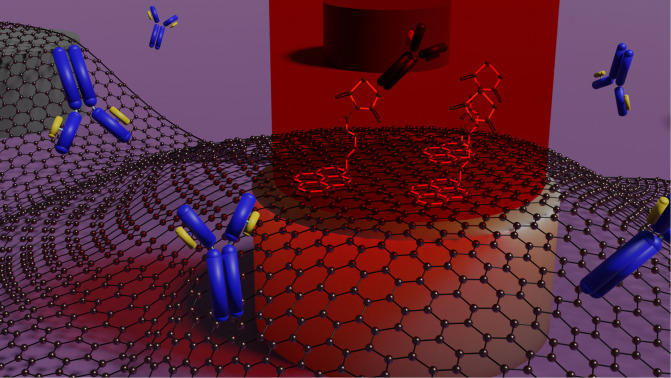

Optical sensing techniques play a crucial role in contemporary biomedical research due to their capability for label-free detection of analytes in real time.^1^ Usually, such techniques work *via* optical resonances that enhance light–matter interaction at certain wavelengths so that the optical response changes dramatically when the substance to be detected is present. The optical response can be either a shift in the resonant wavelength in the case of refractomeric sensing^2^ or the appearance of scattering peaks associated with vibrational resonances of the analyte for Raman biosensing.^3^ In particular, Raman scattering is an interesting optical sensing technique because it provides molecule-specific information without requiring labels or chemical markers. Optical resonances can increase the efficiency of the Raman scattering process. In particular, the use of plasmonic-character resonances in metallic nanostructures, with their associated electromagnetic hot-spots in subwavelength regions, has enabled the technique called surface-enhanced Raman spectroscopy (SERS).^4^ However, metallic nanostructures display several relevant drawbacks with regard to practical applications. First, they suffer from large absorption losses due to the light–metal interaction.^5,6^ Moreover, the illumination of a nanostructured metallic substrate increases its temperature, which can affect the performance of the device and cause degradation of the analyte.^7,8^

High-index nanostructured dielectric substrates offer a solution to such limitations and, as a result, may expand the range of applications of optical sensors. Interestingly, these structures ensure quite high local field enhancements with minimal thermal effects,^9^ which can be of particular interest for application as a SERS substrate.^10^ Moreover, they are also compatible with standard semiconductor nanofabrication techniques, which is not the case with plasmonic metals such as gold and silver. The underlying principles governing the optical resonances in dielectric subwavelength resonators predominantly originate from their Mie scattering properties, including the excitation of electric and magnetic character resonances.^11–13^ Remarkably, magnetic modes may provide higher Raman enhancement compared to their electric counterparts.^14^ The robust Mie-type resonances observed in high-index dielectric nanostructures, together with the minimal inherent optical absorption losses, collectively contribute to a strong light–matter coupling in tiny hot-spots, usually also accompanied by relatively large optical *Q* factors.^15–17^

Interestingly, the localized electromagnetic hot-spots at the boundaries of the high-index dielectric resonators can significantly enhance the Raman response when a material, such as a two-dimensional (2D) layer, is positioned at these regions.^18,19^ Furthermore, the integration of 2D materials with high-index nanostructured substrates has the potential to enhance the sensitivity of the device, a critical parameter in the performance of high-efficiency optical biosensors.^20^ Among the plethora of available 2D materials, graphene, renowned for its stable chemical properties, exhibits numerous advantages. For example, its synthesis is well established through both top-down and bottom-up approaches, enabling its reliable and reproducible production.^21,22^ Moreover, graphene can be synthesized over large areas with high crystalline quality and minimal defects, making it well-suited for scalable device integration. Finally, the high density of functional sites and strong π–π interactions make graphene a promising platform for the stable immobilization of biomolecules. Consequently, when integrated with high-index nanostructured dielectric substrates, a hybrid dielectric–functionalized graphene SERS platform can be engineered to maximize light–matter interaction upon illumination, combining the chemical versatility of graphene with the optical field confinement enabled by Mie optical resonances.

Graphene functionalization with specific molecular linkers has been shown to enable the selective detection of targeted biomarkers.^23,24^ Among the various mechanisms of graphene functionalization, the incorporation of organic molecules is one of the most widely used.^25^ The honeycomb structure of graphene exhibits affinity for polycyclic aromatic hydrocarbons (such as pyrene), allowing the formation of non-covalent interactions between graphene and molecules containing these functional groups.^26,27^ PBASE is an organic heterobifunctional linker composed of an ester group and a pyrene moiety, as can be seen in Fig. 1 a. As mentioned, the pyrene unit is responsible for graphene functionalization, while the ester group enables covalent bonding to molecules presenting primary amines, thus acting as the sensing component.^28^ In this type of graphene-based sensor, the detection of biomolecules is achieved by monitoring shifts in the Raman 2D band, which reflects changes in the electronic properties and strain state of graphene induced by molecular binding at its surface.

**Fig. 1:**
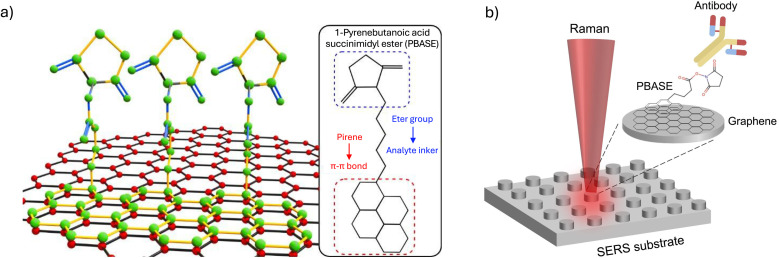
(a) Molecular representation of PBASE molecules interacting with the graphene surface through π–π stacking. The inset shows the molecular structure of PBASE, highlighting the pyrene group responsible for graphene attachment and the succinimidylester group for covalent bonding to the antibody. (b) Schematic illustration of the hybrid SERS platform based on an array of silicon nanopillars on silica coated with a graphene monolayer functionalized with PBASE.

In addition to its application as a biosensing molecule, functionalization using PBASE has also proven to induce p-type doping in graphene, and hence, it can be used to enhance its electrical properties.^29^ Conversely, the aromatic molecule needs to be dissolved using an organic solvent —usually methanol (CH_3_OH) or dimethyl formamide (DMF)—, both reporting an n-doping effect of graphene. This results in a competitive doping between the p-doping of the PBASE molecule and the n-doping effect caused by the electron donor atoms of methanol (oxygen) or the atoms of the organic solvent in DMF (nitrogen).^30^

Despite the challenges posed by competitive doping in the functionalization process, the heterobifunctional nature of PBASE facilitates the detection of a diverse range of biomarkers, including prolactin (PRL) hormone antibodies—a molecule which is involved in reproduction, metabolism, and cancer. Conventional PRL detection relies on blood tests, requiring extensive sample preparation,^31–33^ which highlights the need for sensitive and rapid sensing platforms. In this regard, there is a necessity to fabricate sensitive sensors that allow rapid PRL detection. Similarly, recent pandemics emphasized the need for rapid and accessible diagnosis of viruses, such as SARS-COV-2.

Here, we present a versatile, low-power SERS sensor implemented on a hybrid dielectric–graphene nanostructured substrate for antibody detection (Fig. 1b). The dielectric–graphene sensor, consisting of a functionalized graphene on a silicon nanopillar array on a silica substrate, was used to selectively detect PRL and SARS-CoV-2 antibodies. These analytes were selectively conjugated to the PBASE molecule, which is attached to the graphene *via* a functionalization process using ethanol, as it offers a more stable and less competitive doping environment compared to methanol. Atomic force microscopy (AFM) and Raman spectroscopy techniques were used to characterize the sensor. Finally, we demonstrated through Raman spectroscopy that the doping introduced by PBASE-ethanol functionalization, along with the strain induced by the lithographically patterned surface, does not hinder the detection of biomolecules *via* the 2D Raman peak of graphene. Our results suggest that this optical sensor can operate efficiently at laser powers as low as 2 mW, ensuring minimal energy consumption.

## 1 Methods

### 1.1 Numerical modelling of the silicon nanopillar array

The numerical study of the silicon nanopillars was carried out using the finite integration (FIT) technique module of the commercial 3-D full-wave solver CST Studio Suite®. We considered a single silicon nanopillar placed on a silica substrate, using the refractive indices that the software utilizes for such materials. Perfectly-matched layers (PML) were used at the boundaries of the simulation domain to ensure no reflections. We performed two types of simulations, corresponding to the cases of excitation and collection, in order to estimate the two key processes in Raman scattering.^34^ In excitation mode, we illuminate from the top using a plane wave and monitor the intensity enhancement on top of the silicon nanopillar, which is where the graphene sheet will rest. In the collection mode, we place a horizontal electric dipole on top of the nanopillar and simulate how it radiates light.

### 1.2 Fabrication of silicon nanopillar arrays

6-inch Silicon-on-Insulator (SOI) wafers, with a 220 nm thick silicon, were diced into 30 mm × 20 mm pieces. Next, the diced SOI substrates were cleaned using the following procedure: they were rinsed with running deionized water for 30 s to remove any particles due to the dicing process. Afterwards, they were blown dry in N_2_ and immersed in acetone for 300 s at room temperature (RT). Finally, the diced SOI substrates were sonicated in isopropyl alcohol (IPA) during 300 s at RT and blown dry again with dry N_2_. Using a fluorine-based process, the 220 nm thick silicon layer was etched down to 140 nm with inductively coupled plasma-reactive ion etching (ICP-RIE; STS multiplex, SPTS Technologies Ltd). Afterwards, a 100 nm thick HSQ (Dow-Corning) resist was spun on the 180 nm thick silicon.

The silicon pillars were then defined by electron-beam lithography (Raith 150, Raith GmbH). On the 30 mm × 20 mm sample, 28 frames of 50 µm × 50 µm were patterned. Centered in each frame, which are employed to facilitate the optical characterization of the chip, arrays of 38 s × 38 single silicon nanopillars with a diameter of 130 nm (mean, SEM) and a height of 141.9 ± 4.7 nm (mean, AFM), with a 1 µm period were patterned in the same lithography step (see SI Fig. S1 and 2a).

**Fig. 2:**
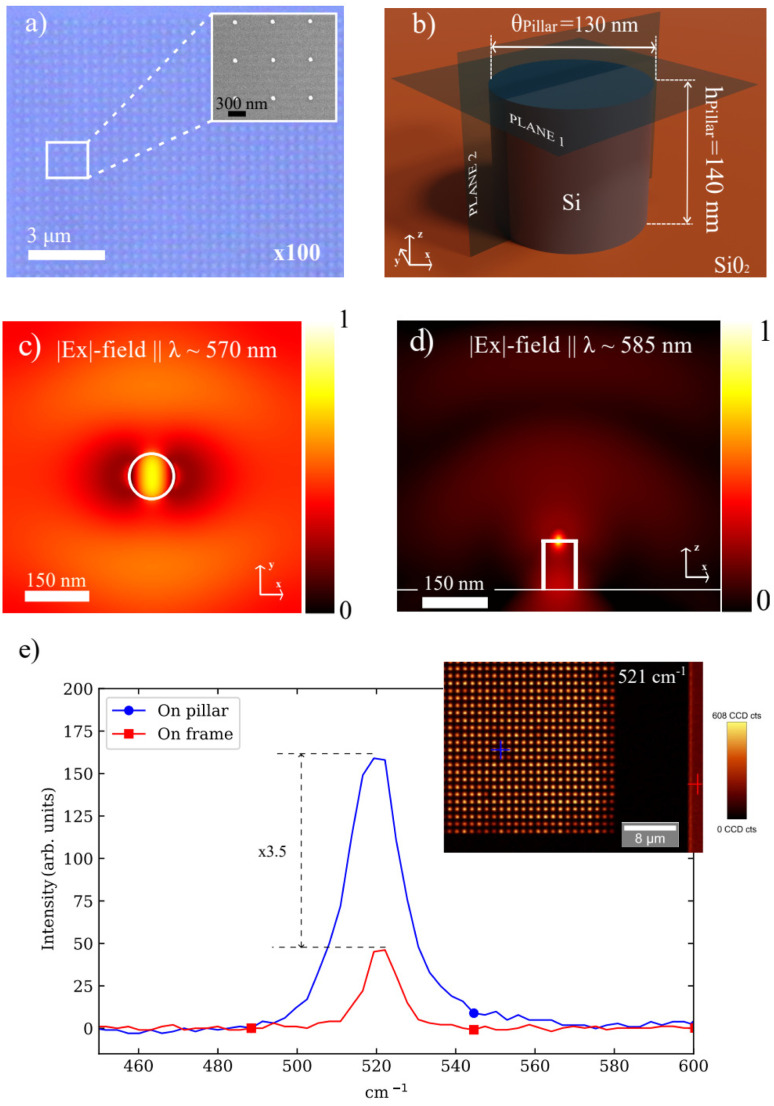
Simulation and characterization of the silicon nanopillars. (a) Optical micrograph of the fabricated silicon nanopillar array on the SOI substrate. The inset shows a scanning electron microscopy (SEM) image of the periodic nanopillars with a diameter of approximately 130 nm and a 1 *µ* m pitch. (b) Schematic representation of a single silicon nanopillar unit cell used in simulations, with a disk diameter (*θ*_Pillar_ = 130 nm) and a height (*h*_Pillar_ = 140 nm) on a SiO_2_ substrate. The electromagnetic field monitors were placed on the planes shown. (c) *Z*-plane electric field on top of the silicon nanopillar showing how the EM field is confined on the nanopillar surface. (d) *Z*-plane electric field in the middle of the nanopillar where the propagation of the emitted field is shown. (e) SERS induced by the nanopillar. The blue line corresponds to the field intensity on the top of one nanopillar highlighted with a blue cross in the inset image whereas the red line shows the spectrum on the silicon frame that surrounds the area with the silicon nanopillar array. The inset image is a Raman image of the resonator substrate of the 521 cm^−1^ crystalline Si vibrational mode.

### 1.3 Graphene transfer and functionalization with PBASE

Two different sources of graphene were utilized: chemical vapor deposition (CVD) commercial 10 mm × 10 mm “Easy Transfer” graphene (Graphenea), and graphene transferred from a highly ordered pyrolytic graphite (HOPG) substrate using an all-dry transfer method (see Fig. S1c).^35^ The CVD monolayer graphene was transferred using the transfer process described in the commercial data sheet (https://www.graphenea.com): immersing the sample for one hour in acetone (50 °C), followed by 1 h in IPA at room temperature. Conversely, a polydimethylsiloxane (PDMS) stamp-assisted soft lithography method was employed for the deposition of exfoliated graphene using a home-made system.^36^ The graphene functionalization (for both CVD monolayer graphene and mechanically exfoliated graphene flakes) was carried out using a 2 mM PBASE solution (30.8 mg) dissolved in absolute ethanol (40 ml). The samples were immersed in the solution for 60 min (RT), then immersed in absolute ethanol and sonicated for 5 min, rinsed with absolute ethanol and blown dry with N_2_.

### 1.4 Inmobilization of antibodies

10 µl of drops were poured onto the fabricated nanostructures and cured during 2 h at room temperature, followed by washing with phosphate-buffered saline (PBS) and drying with N_2_. Two different antibody concentrations were used, depending on the specific antibody:

• SARS-CoV-2 Spike S1 antibody: 200 mM solution of PBS and the SARS-COV-2 Spike S1 antibody, Rabbit MAb 1 mM, #40150-R007, SinoBiological.

• Prolactin: 40 mM solution of PBS and a mouse monoclonal anti-prolactin antibody 200 mM, Mouse Anti-PRL antibody, sc-46698, Santa Cruz Biotechnology.

### 1.5 Characterization

#### 1.5.1 Raman spectroscopy measurement

Raman spectroscopy was performed at room temperature. A confocal Raman imaging microscope (alpha 300R, WITec) was employed in the backscattering configuration using a 100× objective and a 600 g mm^−1^ grating with 2.8 cm^−1^ spectral resolution. The excitation energy (wavelength) from the laser diode module was 2.33 eV (532 nm). The 20 µm × 20 µm scans (150 points per line, 150 lines per image, and 0.1 s integration time) at 2 mW were carried out to measure the matrix of the fabricated silicon nanopillars. The D, G, D′, and 2D Raman fingerprint bands of graphene were fitted using a Lorentzian function with the FitRaman software.^37^ Information about the peak position, full width at half maximum (FWHM), intensity, and area was then retrieved.

#### 1.5.2 Atomic force microscopy measurements

The Atomic Force Microscopy (AFM) measurements were performed using silicon AFM probes (PPP-NCHR-50, NANOSENSORS™, NanoWorld AG) with an alpha 300R (Witec) microscope. Tapping-mode measurements (*f*_r_ = 300 kHz; *K* = 300 N m^−1^) were carried out to evaluate the fabrication and functionalization processes. All AFM images were processed with the WSxM software from Nanotec Electronica S.L.^38^

## 2 Results

### 2.1 Numerical design of the silicon nanopillars

High-index dielectric nanoparticles can support a variety of multipolar resonances whose interference can lead to a large field enhancement in the particles as well as in their surroundings, which is useful for enhancing light–matter interaction. Moreover, dielectric nanoparticles can be designed so that the far-field scattering of the excited multipoles interferes destructively in the far-field,^39^ resulting in the so-called anapole states, which have been proven to increase the efficiency of Raman scattering.^40^ To explore and optimize these resonant effects in our system, we performed electromagnetic simulations using finite-difference-time-domain (FDTD) with CST Microwave Studio software for isolated silicon nanopillars and an hexahedral mesh with 20 cells per wavelength. The simulations were carried out in a dedicated server with 64 GB and an Intel Xeon E-2126 G processor. Note that although the nanopillars were fabricated forming arrays to facilitate the experiments, the separation is much larger than the wavelengths used in the experiments, meaning that they are electromagnetically decoupled. The simulations were performed for an isolated pillar (period ≫ *λ*, negligible coupling). A parameter sweep of the measured diameter 130 ± 10 nm and *h*_DISK_ = 140 ± 5 nm in height changes the calculated local-field/Raman enhancement at 532 nm by 15–20%. The morphology of the fabricated samples was characterized using optical microscopy, scanning electron microscopy (SEM) (see Fig. 2a), and AFM to use realistic parameters in the numerical simulations. The individual nanopillars exhibited well-defined dimensions, with an average diameter of *θ*_Pillar_ = 130 nm, as determined by SEM, and a height of *h*_Pillar_ = 140 ± 2 nm measured by AFM (see SI Fig. S1b). We considered that the nanopillars were placed on a SiO_2_ substrate (see the sketch in Fig. 2b). To model the performance of the nanopillar in excitation, we illuminated the pillar with a plane wave incident from above at *λ* = 570 nm (corresponding to the wavelength of the Raman laser), as in the experiments, and calculated the *E*_*x*_ on the plane placed on top of the disk (*z* = 140 nm). Fig. 2c represents the field intensity (*E*_*x*_^2^) normalized to its maximum value on that plane. The field map clearly shows an intensity hot-spot on top of the disk, indicating that Raman centers placed there will be strongly excited by the incoming plane wave. To test the collection performance, we placed an electric dipole oriented along the *x*-axis on top of the disk and on its center, tuned at a wavelength corresponding to one of the expected Raman scattered signals (*λ* = 585 nm). As shown in Fig. 2d, the dipole radiation is enhanced by the hot-spot, and a large radiation in the vertical direction is observed, meaning that the signal produced by the Raman centers will be enhanced by the local hot spot and efficiently collected by the Raman spectrometer used in the experiments.

### 2.2 Raman characterization of silicon nanopillar arrays

Crystalline silicon exhibits a prominent and well-defined primary Raman peak—the transverse optical (TO) mode—located at 521 cm^−1^, which arises from the vibrational motion of silicon atoms in the crystal lattice. To validate the SERS performance of our dielectric platform, this characteristic Raman resonance at 521 cm^−1^ was employed. The enhancement factor *E*, defined as *E* = 〈*S*_on_〉/〈*S*_out_〉, where *S* refers to the peak intensity, was calculated from the normalized Raman spectra of silicon measured on top of the nanopillars (*S*_on_) and outside the nanostructured region on the silicon frame surrounding the nanopillar array (*S*_out_). As shown in Fig. 2e, the Raman spectrum recorded directly on top of a silicon nanopillar exhibits an intensity enhancement of three times as compared with the signal measured outside the region with the nanopillar array. This pronounced enhancement demonstrates the strong local field enhancement provided by the dielectric structure. The inset image shows a Raman intensity map at 521 cm^−1^ across the nanopillar array. The brightest spots in the image correspond to the locations of the silicon nanopillars, further confirming the spatially localized SERS effect induced by the nanostructured dielectric surface.

### 2.3 Prolactin and SARS-COV-2 sensor

**Fig. 3:**
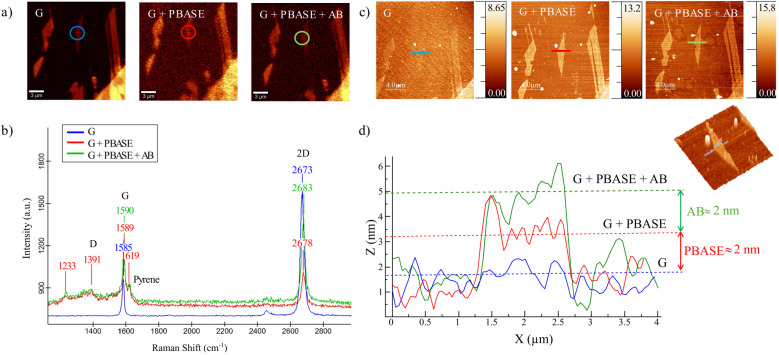
Raman spectroscopy characterization. (a) Raman images of the graphene G band (1575 cm^−1^), obtained for the three different configurations. (b) Raman spectra of graphene (G, blue), graphene functionalized with PBASE (G + PBASE, red) and graphene functionalized with PBASE and the particular antibody (G + PBASE + AB, green). (c) AFM Topography images (20 µm × 20 µm), obtained for the three different configurations. (d) AFM profiles of graphene (green), graphene functionalized with PBASE (red), and functionalized with PBASE and the particular antibody (blue).

Having confirmed the SERS enhancement capabilities of the dielectric nanopillars through the characteristic Raman response of silicon, our focus shifts to graphene integration. This section provides a detailed description of graphene functionalization with PBASE molecules, a process considered essential to enable selective biomolecular detection on the hybrid SERS platform. As mentioned in the Methods section, the functionalization with the PBASE was first developed using Scotch Tape exfoliated graphene from HOPG high-quality crystals. In this scope, AFM and Raman spectroscopy measurements were carried out to probe the correct PBASE functionalization. Fig. 3 presents the characterization of graphene at different functionalization stages: pristine graphene (G), graphene functionalized with PBASE (G + PBASE), and graphene functionalized with both PBASE and a specific antibody (G + PBASE + AB). Few graphene flakes were obtained *via* dry mechanical exfoliation and transferred onto a silicon substrate with a layer of 300 nm of SiO_2_ for further characterization by Raman spectroscopy and AFM. These flakes were specifically selected due to their high quality, flat surfaces, and minimal edge features, which made them ideal for confirming the presence of PBASE and the antibody at each stage of the functionalization process *via* AFM. Fig. 3a shows the Raman intensity maps (20 µm × 20 µm scans) corresponding to the G band (around 1575 cm^−1^) of several graphene flakes for the three different configurations (for full Raman characterization, see intensity maps in Fig. S2). A gradual decrease in G band intensity is observed in Fig. 3b, corresponding to Raman spectra of the same graphene flake (marked with colored circles in Fig. 3a) at each functionalization step. This decrease may indicate electronic interactions with adsorbed molecules that quench the Raman signal or alter the local electronic environment of the graphene. Upon functionalization with PBASE, new peaks emerge, such as the one around 1233 cm^−1^, attributed to the pyrene group in PBASE. A slight broadening and shift of the G band (from 1585 cm^−1^ to 1590 cm^−1^) is also evident, indicating π–π stacking interactions between PBASE and the graphene lattice. The 2D band (around 2680 cm^−1^) decreases in intensity after antibody binding, possibly reflecting changes in the doping level or strain within the graphene sheet due to the presence of biomolecules. Additional weaker peaks in the G + PBASE + AB spectrum support the successful immobilization of the antibody (additional full Raman and optical characterization of mechanically exfoliated graphene flakes is presented in SI S3).

A shift of the 2D peak is also evident after functionalization that would indicate a modification of the electronic environment of graphene, likely due to charge transfer or induced strain from the attached molecules.^41^ These effects will be further discussed in the next sections. Fig. 3c presents AFM topography images (20 µm × 20 µm scan) of the same sample at the same area inspected by Raman spectroscopy. Fig. 3d shows the height profiles extracted from the AFM images at a single flake of graphene at every stage of the functionalization process. A clear increase in height is observed across the three stages: approximately 1 nm for bare graphene (green), 3 nm for G + PBASE (red), and 2 nm for G + PBASE + AB (blue). These increases in thickness quantitatively confirm the sequential adsorption of PBASE and the antibody on the graphene surface. The final sample, with both PBASE and antibody, exhibits increased roughness and heterogeneous features, which are consistent with the attachment of large biomolecular structures on the surface. The combined Raman and AFM results confirm the successful stepwise functionalization of the graphene surface and validate the use of PBASE with ethanol as a solvent. Ethanol is used in this work instead of methanol or DMF. Due to its lower polarity and weaker electron-donating character, ethanol reduces the competing doping effects, resulting in a more stable and controlled functionalization of the graphene surface. (The effect of ethanol doping is discussed in SI Section S4).

After successfully attaching the PBASE molecule using ethanol, the anti-PRL antibody and SARS-CoV-2 Spike S1 antibody were immobilized to evaluate the fabrication of a reliable biosensor based on the hybrid dielectric–functionalized graphene SERS platform. A commercial graphene stamp was employed for large-area and uniform transfer to ensure the presence of a monolayer of graphene on top of the silicon nanopillar arrays during this process.

**Fig. 4:**
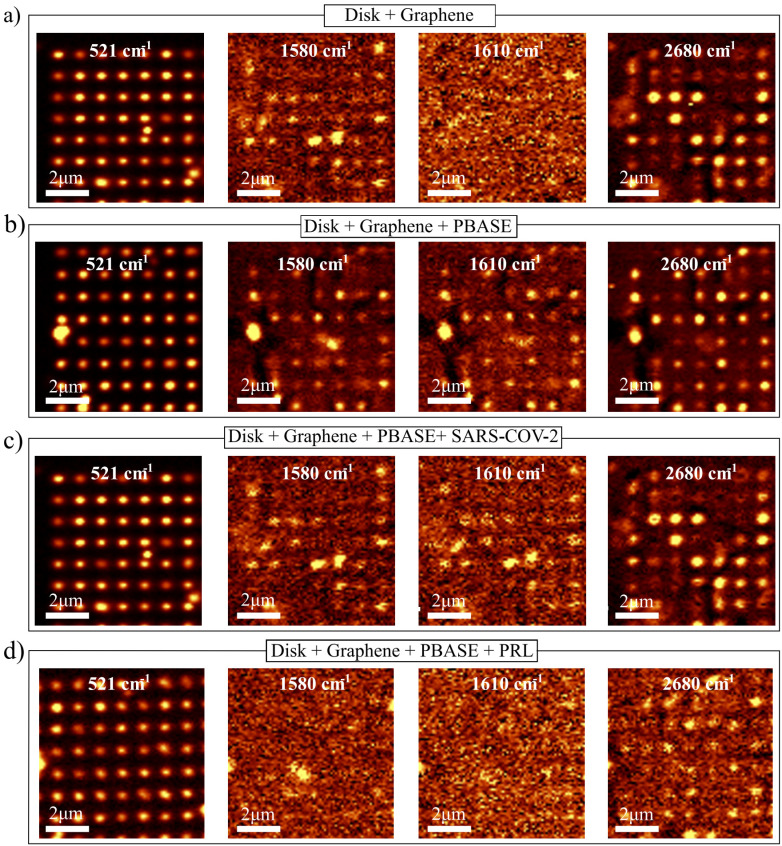
Characteristic Raman resonances of silicon, graphene and PBASE at different stages of the fabrication: (a) dielectric–graphene sensor: dielectric silicon nanopillar + graphene. (b) Functionalized sensor: dielectric silicon nanopillar + graphene + PBASE. (c) SARS-CoV-2 Spike S1 antibody sensor: dielectric silicon nanopillar + graphene + PBASE + SARS-CoV-2 Spike S1 antibody. (d) Prolactin sensor: dielectric silicon nanopillars + graphene + PBASE + prolactin antibody.

The functionalization and the antibody immobilization processes were characterized *via* Raman spectroscopy measurements. Fig. 4 shows Raman intensity maps (10 µm × 10 µm scans) of an array of 30 silicon pillars at characteristic vibrational modes for each stage of sensor functionalization: (a) silicon nanopillars with graphene, (b) graphene functionalized with PBASE, (c) subsequent immobilization of SARS-CoV-2 Spike S1 antibodies, and (d) immobilization of prolactin (PRL) antibodies. The maps at 521 cm^−1^ (left column) correspond to the silicon transverse optical (TO) phonon mode and delineate the location of the nanopillars, confirming consistent SERS enhancement across the nanopillar array throughout all fabrication stages. All Raman maps were acquired at the upper-left corners of matrices fully covered with graphene after the corresponding functionalization process. In this system, the detection mechanism is not based on direct Raman peaks from the biomolecules but is rather based on the spectral shift of graphene 2D band, which sensitively reflects molecular adsorption and charge transfer at the graphene–analyte interface.

The successful transfer of the graphene monolayer and its endurance during the immobilization process is confirmed by the presence of its Raman fingerprint bands: the G band at around 1583 cm^−1^, together with the defect-related bands *i.e.* the D band (1300 cm^−1^), its overtone the 2D band (2680 cm^−1^) and the D′ band (1620 cm^−1^).^42^ (Fig. 4a). The successful attachment of the PBASE molecule on the graphene surface was confirmed by the presence of the Raman peak at 1610 cm^−1^. This peak —associated with the pyrene group resonance—is absent in the pristine graphene (Fig. 4a) but visible after PBASE functionalization (Fig. 4b–d).

Its persistence in (Fig. 4c) and (Fig. 4d) confirms that PBASE remains anchored to the graphene surface after antibody conjugation, together with the appearance of the D and D′ bands of graphene, which have been previously employed to prove the successful attachment of PBASE on the graphene surface.^30,43^

Once the antibodies are immobilized, the Raman signal from graphene becomes attenuated due to the additional layers of PBASE and antibodies covering its surface. However, the signal remains detectable, thanks to the SERS enhancement provided by the silicon nanopillars. Interestingly, minor nanoscale variations (<5%) in pillar geometry introduced during the fabrication of the nanopillars are reflected in the differing intensities observed at 521 cm^−1^, revealing the sensitivity of the platform to subtle structural differences.

**Fig. 5:**
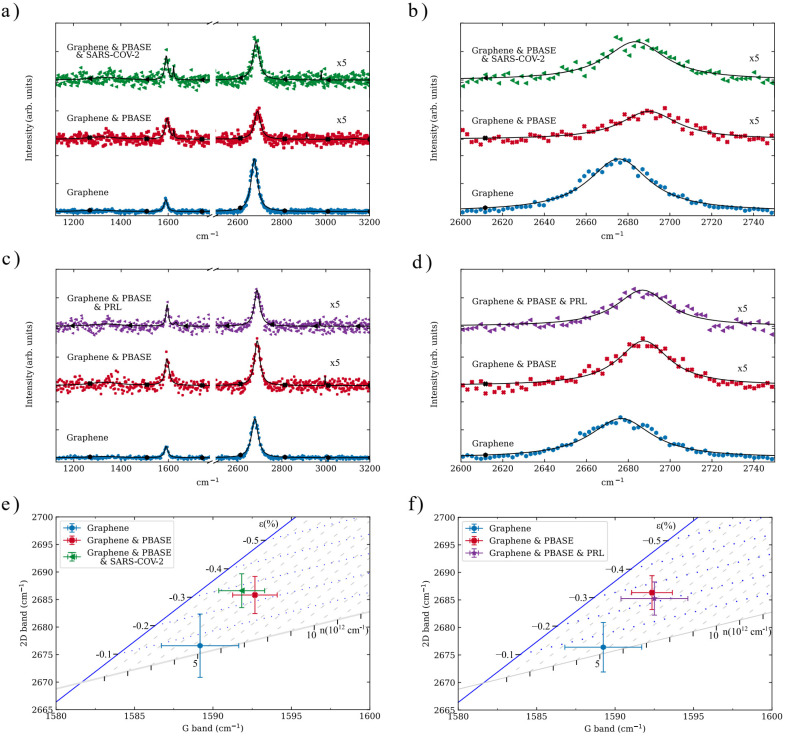
(a) and (c). Raman spectra (scatter) and Lorentzian fitting (line) of a representative silicon pillar at different stages of SARS-COV-2 and PRL antibody immobilization. (b) and (d). Zoomed Raman spectra on the 2D band of graphene showing the shifts at each stage of the immobilization process. (e) and (f). Strain and doping vector decomposition of the antibody immobilization process.

The 30 silicon pillars for each antibody were monitored, extracting their Raman spectra from the Raman mapping scans for statistical purposes. Their spectra were then analyzed (Fig. 5a–d); and the strain and doping contributions were obtained for each antibody (Fig. 5e and f).

The ratio between the 2D and G bands was employed as a measurement of the quality of the graphene. The transferred graphene on the silicon nanopillars used for detecting SARS-COV-2 antibodies exhibited an average *I*_2D_/*I*_G_ ratio of 3.6 ± 0.8, and an *I*_2D_/*I*_G_ ratio of 3.9 ± 0.9 was observed for the substrates used for PRL antibody detection. Following the graphene functionalization using the PBASE molecule, the *I*_2D_/*I*_G_ ratios were reduced to 1.6 ± 0.5 and 1.6 ± 0.5, respectively, indicating an increased disorder in the graphene layer.

The displacement of the G and 2D bands is indicative of doping and strain within the graphene layer.^44^ Specifically, the 2D band exhibits greater sensitivity to strain compared to the G band due to its larger Grünesien parameter.^45^ This relative displacement, which is similar for both PBASE functionalizations, can be observed when the PBASE is attached to the graphene layer. In this case, the 2D band undergoes a blue shift of approximately 10 cm^−1^ from its position in the single graphene layer (2676 ± 4 cm^−1^).

This shift is 3 times larger than those previously reported by Nguyen *et al.*^43^ In comparison, their values are reported for a single-layer graphene, whereas in our study, the spatial resolution of the Raman microscope (350 nm) provides an average information of the silicon nanopillar and its surroundings. Consequently, the observed displacement can be indicative of the strain exerted on our graphene layer, as it is positioned on the silicon pillar with the PBASE molecule attached to its surface.

One of the key aspects of ensuring the presence of a monolayer graphene sheet is that the contributions of strain and doping can be inferred from Raman spectroscopy measurements applying the vector decomposition method suggested by Lee *et al.*^46^ In this representation, the space of frequencies of the G and 2D bands is divided by unit vectors representing the strain and the hole doping effects (Fig. 5).

The transferred graphene outside the silicon pillars exhibits compressive strain (*ε*_Si_ = −0.15 ± 0.03%). However, on the silicon pillars, this strain is compensated due to the tensile stress induced by the substrate, resulting in initial strain values of *ε*_0_ = −0.04 ± 0.14% for the SARS-CoV-2 sensor and *ε*_0_ = −0.03 ± 0.10% for the PRL sensor (Table 1). After the PBASE attachment, the compressive strain increases for both sensors—*ε*_PBASE_ = −0.23 ± 0.09% (SARS-COV-2) and *ε*_PBASE_ = −0.25 ± 0.10% (PRL)—because of the deformation exerted on the graphene layer. Once the antibody immobilization is carried out, the strain distribution varies: for the SARS-CoV-2 Spike S1 antibody, the compressive strain further increases (*ε*_AB_ = −0.27 ± 0.09%), whereas the prolactin antibody slightly reduces the strain experienced by the graphene layer (*ε*_AB_ = −0.22 ± 0.08%).

Outside of the region with the nanopillar arrays, our graphene experiences a p-doping *n*_init_ = 6.25 ± 0.12 × 10^12^ cm^−2^ (Table 2), probably influenced by the solvents employed during the transfer method. Significantly, the doping level of the graphene on the silicon pillars is reduced—*n*_pillars_ = 4.5 ± 2.2 × 10^12^ cm^−2^ for the SARS-COV-2 sensors, and *n*_pillars_ = 4.6 ± 1.8 × 10^12^ cm^−2^ for the PRL sensor. This reduction is caused by the charged surface states and impurities of SiO_2_, which reduce the electrical response of graphene when placed on its surface.^47,48^ After the PBASE functionalization, the doping level is further reduced to *n*_PBASE_ = 3.8 ± 1.8 × 10^12^ cm^−2^ for the SARS-COV-2 sensor and to *n*_PBASE_ = 3.2 ± 2.2 × 10^12^ cm^−2^ for the PRL sensor. The effect of both antibodies on the doping levels is different: whereas the SARS-CoV-2 Spike S1 antibody further reduces the doping of graphene (*n*_AB_ = 2.6 ± 1.8 × 10^12^ cm^−2^), the PRL antibody induces a slight increment of negative charges (*n*_AB_ = 3.9 ± 2.1 × 10^12^ cm^−2^).

**Table 1:** Strain exerted on graphene during the immobilization of antibodies

Strain (*ɛ* (±) (%))				
On the silicon surface		Transfer	PBASE	Antibody
−0.15 (0.03)	SARS-CoV-2	−0.04 (0.14)	−0.23 (0.10)	−0.27 (0.09)
	PRL	−0.03 (0.10)	−0.25 (0.10)	−0.22 (0.08)

**Table 2:** Doping levels experienced by the graphene layer during the immobilization of antibodies

Doping (*n* (±) (10^12^ cm^−1^))				
On the silicon surface		Transfer	PBASE	Antibody
6.25 (0.12)	SARS-CoV-2	4.5 (2.2)	3.8 (1.8)	2.6 (1.8)
	PRL	4.6 (1.8)	3.2 (2.2)	3.9 (2.1)

The demonstration of molecular discrimination in our hybrid dielectric–graphene sensor is based on three complementary observations: (i) the distinct strain and doping responses extracted *via* Raman vector decomposition (Fig. 5e and f) for the two antibodies (SARS-CoV-2 and PRL), (ii) the opposite trends in charge carrier concentration after antibody immobilization (Table 2), and (iii) the different Raman 2D-band shifts observed for each case, with a stronger compressive strain for SARS-CoV-2 and partial relaxation for PRL. This combination of spectral and electronic fingerprints constitutes molecular discrimination at the proof-of-concept level. Therefore, the sensor's molecular discrimination capability is evidenced by the distinct Raman responses obtained for the two antibodies. As shown in Fig. 5e and f and Tables 1 and 2, the graphene 2D and G bands display clearly differentiated strain and doping trends: the SARS-CoV-2 Spike S1 antibody induces a stronger compressive strain (*ε* = −0.27 ± 0.09%) and a reduction in carrier density (*n* = 2.6 × 10^12^ cm^−2^), while PRL causes a slightly lower strain (*ε* = −0.22 ± 0.08%) and an increase in doping (*n* = 3.9 × 10^12^ cm^−2^). These opposite strain–doping trends confirm that the dielectric–graphene platform can distinguish between different biomolecular interactions through their specific perturbations of the graphene Raman response.

## 3 Conclusions

In conclusion, we have experimentally demonstrated the feasibility of using dielectric SERS substrates containing arrays of silicon nanopillars decorated with graphene to detect prolactin as well as SARS-CoV-2 Spike S1 antibodies. Because of the enhancement of the Raman effect induced by the resonant response of the silicon nanopillars, low-power and short integration time measurements can be taken, hence reducing the possibility of burning or degradation of the analyte. Beyond demonstrating the biosensing capabilities of our hybrid optical device, we performed a detailed investigation of how both lithographically induced strain and molecular doping influence the Raman response of graphene. Our analysis, based on vector decomposition of the G and 2D Raman bands, reveals that the local strain introduced by the patterned dielectric substrate can shift the graphene vibrational modes significantly, yet predictably, without hindering its sensing performance. The opposite strain and doping trends observed for the two antibodies demonstrate the sensor capability for molecular discrimination, providing direct experimental evidence that the graphene layer acts as a sensitive transducer of specific biomolecular interactions on the dielectric nanopillar substrate. Additionally, we show that ethanol-mediated PBASE functionalization induces controlled p-type doping while minimizing the competing effects typically observed with other solvents such as methanol or DMF. These findings highlight the robustness of the platform and underscore the importance of understanding and controlling both mechanical and electronic perturbations when designing graphene-based SERS sensors for biochemical detection. Notably, our method could be applicable to detect other substances using the same kind of photonic structure, which could be potentially manufactured in large volumes and at low cost using standard silicon fabrication tools.^49^ A quantitative limit-of-detection analysis, involving concentration-dependent calibration, lies beyond the scope of the present study and will be addressed in future work to further evaluate the sensing capabilities of the platform. Nevertheless, we envision that this approach could ultimately enable the development of disposable SERS chips for point-of-care biosensing and remote detection of chemical or biological substances.

## Author contributions

A. M. and E. P. C. conceived and led the work. V. J. G. and M. S. L. led and supervised CVD graphene processing. M. S. L. performed the Raman analysis and evaluated strain and doping effects. J. R., M. C. P., I. G. L., S. Z., E. Z., and M. A. conducted the experimental work. E. D. E. and J. R. carried out the CST simulations. J. R., M. S. L., M. C. P., and E. P. C. wrote the manuscript, and all authors contributed to its review and revision. All authors contributed intellectually to the development of the work.

## Conflicts of interest

There are no conflicts to declare.

## Supplementary Material

NA-008-D5NA00847F-s001

## Data Availability

The datasets supporting this article are openly available in Zenodo at https://zenodo.org/records/17023716. Supplementary information (SI): S1 includes information of the structural characterization of the dielectric SERS substrate. S2 and S3 provide more Raman spectroscopy measurements of the functionalization process. S4 provides information on the Raman enhancement factor. See DOI: https://doi.org/10.1039/d5na00847f.
