# Supplementary material for: Hybrid dielectric–graphene nanostructured SERS substrates for antibody sensing
Source: Nanoscale Adv. 2025 Nov 18;8(3):835–45. doi: 10.1039/d5na00847f (PMC12684743; doi:10.1039/d5na00847f)
Supplement: NA-008-D5NA00847F-s001 [file NA-008-D5NA00847F-s001.pdf]

Supplementary Information (SI) for Nanoscale Advances.
This journal is © The Royal Society of Chemistry 2025

Supplementary Information:

Hybrid Dielectric-Graphene SERS metasurfaces for anti- body sensing

Javier Redolat,[a,‡] Miguel Sinusia Lozano,[a,‡] María Camarena Pérez,[a,b] Ignacio González-Llácer,[a] Sofiya Zorina,[a] Eva Zafra,[a] Mar Alonso Chornet,[a,c] Evelyn Díaz-Escobar,[a] Víctor J. Gómez,[a] Alejandro Martínez,[a] and Elena Pinilla-Cienfuegos*[a]

[a] Nanophotonics Technology Center (NTC), Universitat Politècnica de València, Valencia, Spain. Tel: +34 963 87 97 36; E-mail: epinilla@ntc.upv.es

[b] Department of Microelectronics, Faculty of Electrical Engineering, Mathematics and Computer Science, Delft University of Technology, Delft, The Netherlands.

[c] École des Mines de Saint-Étienne. Saint-Étienne, France.

Supplementary Information S1. Structural characterization of the fabricated dielectric SERS substrate.

Silicon-on-Insulator (SOI) wafers with a 220 nm-thick silicon device layer were diced into 30 mm × 20 mm substrates. The silicon layer was subsequently thinned down to 140 nm using an inductively coupled plasma reactive ion etching (ICP-RIE) process, and a 100 nm-thick HSQ resist layer was spin-coated onto the etched surface. Silicon nanopillars were defined by electron-beam lithography. Each 30 mm × 20 mm substrate contained 28 patterned Si frames of 50 µm × 50 µm to facilitate optical characterization of the SERS substrate. At the center of each frame, arrays of 38 × 38 silicon nanopillars with a 1 µm period were fabricated in the same lithography step. The resulting nanopillars exhibit an average height of 141.9 ± 4.7 nm and a width of 130 nm, as confirmed by AFM and SEM measurements (Table S1 and Figure S1), corresponding to a ~3% structural dispersion, a typical value for e-beam lithography and ICP-RIE processes.

Pillar (Row)	C1	C2	C3	C4	C5
**P1**	145	148	148	147	147
**P2**	146	144	146	146	146
**P3**	144	144	141	144	144
**P4**	140	144	142	139	134
**P5**	140	143	140	137	132
**P6**	137	142	139	137	130

Table S1: Measured nanopillar heights (in nm) obtained from AFM analysis of 30 nanopillars shown in Figure S1c. Rows (P1–P6) correspond to nanopillars along one direction (rows) and columns (C1–C5) along the orthogonal direction within the array. The average height is 141.9 ± 4.7 nm.

The 30 silicon nanopillars analyzed correspond to those measured within the 5 μm × 5 μm AFM scan shown in Figure S1b, taken from the upper-left corner of a single 38 × 38 nanopillar array within matrix 6B (Figure S1a). This region was selected so that a single AFM scan could include both a representative number of nanopillars and a flat silicon area in the upper-left corner for reference, providing sufficient accuracy for height measurements and a statistically meaningful sample size to calculate height dispersion. The 30 pillars correspond to all those contained within that scan region and were used to extract the average height and dispersion values (141.9 ± 4.7 nm). The Raman analysis was subsequently performed on 30 nanopillars as well, to ensure consistency between the structural and optical characterizations (region highlighted in Figure S1e).

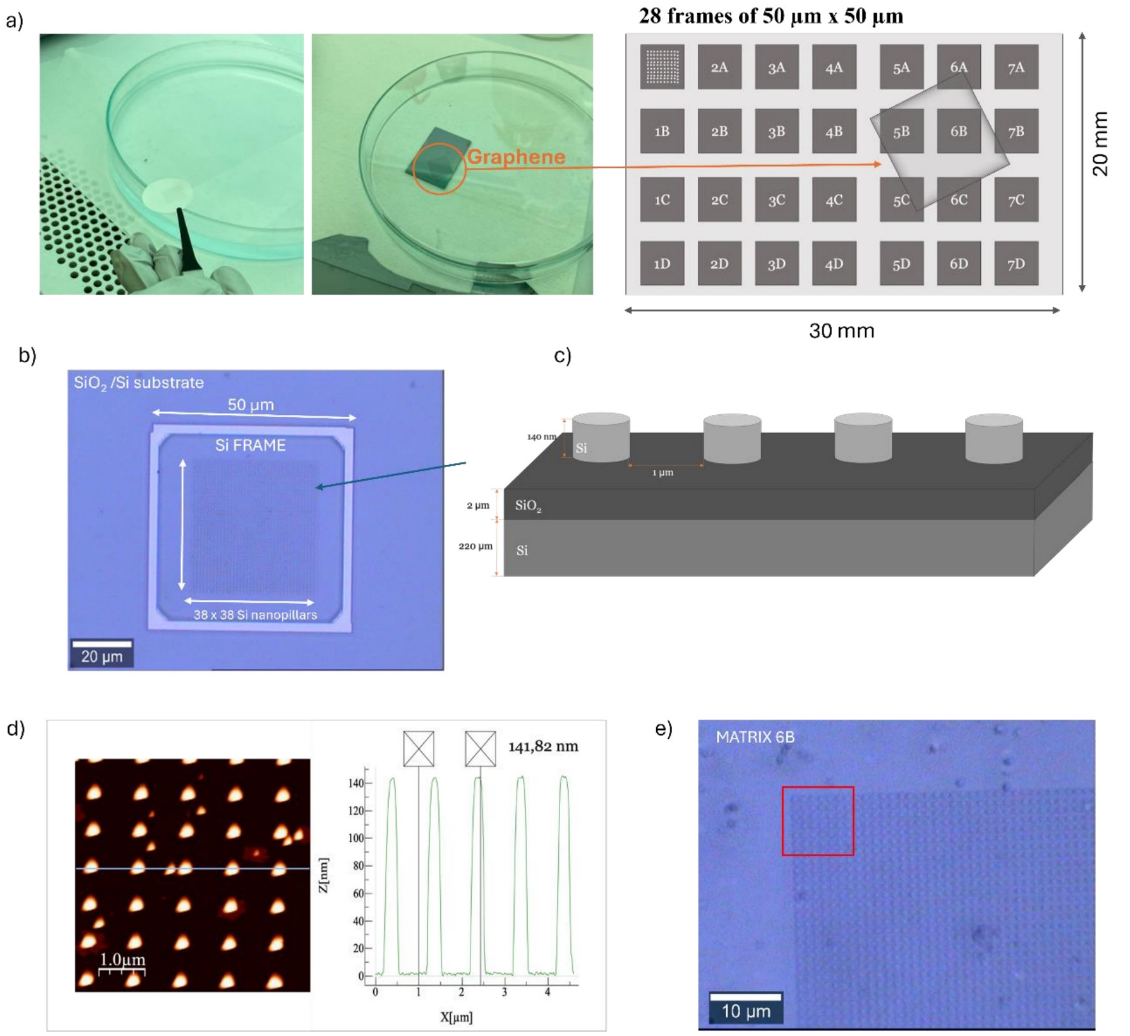

Figure S1. **Structural characterization of the fabricated dielectric nanopatterned SERS substrate**. a) *Left*: Image of the graphene film on a polymer support being positioned onto the nanostructured silicon substrate using tweezers. *Center*: Graphene film aligned over the chip prior to transfer, highlighted in orange. *Right*: Schematic layout of the 30 mm × 20 mm chip showing the 28 patterned Si frames (50 µm × 50 µm each) and the approximate placement of the graphene flake covering multiple functional zones.
b) Optical microscopy image of one 50 µm × 50 µm Si frame containing a 38 × 38 array of silicon nanopillars, surrounded by an unpatterned Si border. *Right*: Cross-sectional schematic of the Si nanopillars (diameter ≈130 nm, height ≈140 nm, period = 1 µm) on the SiO_2/Si substrate.
c) Atomic force microscopy (AFM) topography image of the nanopillar array showing the periodic arrangement with well-defined geometry. The AFM height profile extracted along the indicated line reveals an average nanopillar height of 141.9 ± 4.7 nm, in good agreement with the design parameters. e) Optical microscopy image of matrix 6B after graphene transfer. The red square indicates the region where the Raman and SERS measurements were performed.

Supplementary Information S2. Raman full characterization.

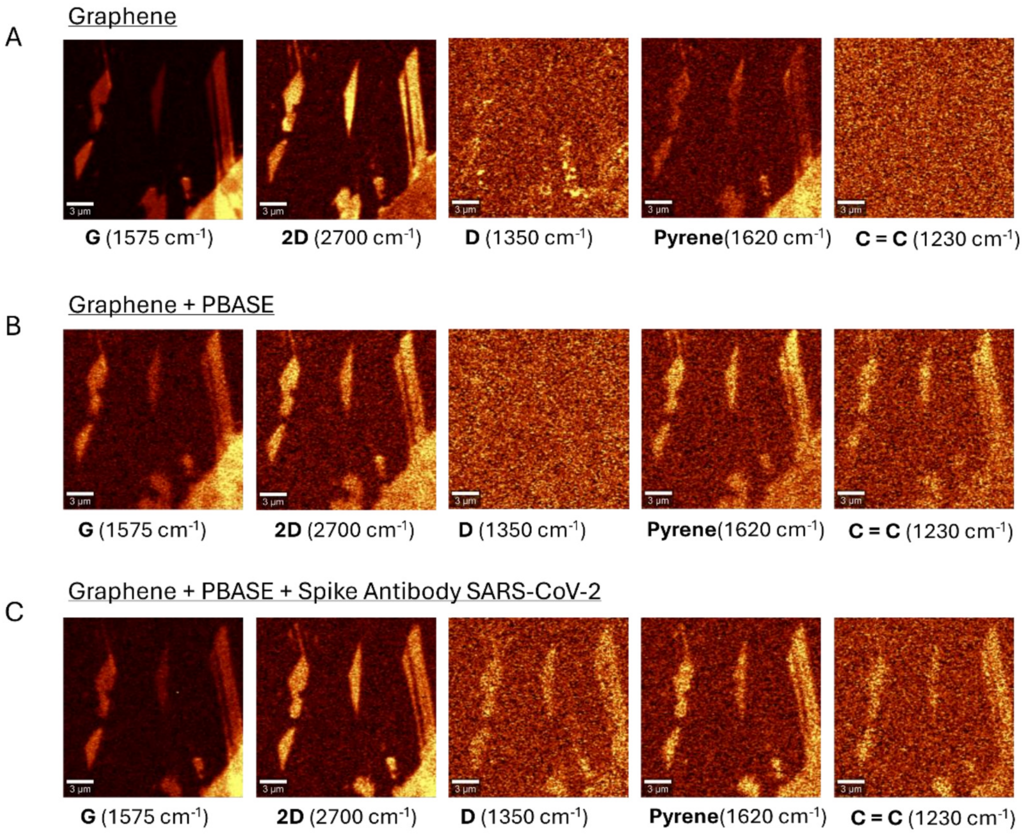

Figure S2. Raman intensity maps of graphene surfaces at different stages of functionalization. Each row corresponds to a specific functionalization condition: a) pristine graphene (G), b) graphene with PBASE (G + PBASE), and c) graphene with PBASE and immobilized antibodies (G + PBASE + AB). Columns represent Raman maps at characteristic Raman shifts: D band (~1350 cm^{-1}), G band (~1580 cm^{-1}), and 2D band (~2670 cm^{-1}), as well as PBASE- and antibody-associated peaks. Progressive functionalization leads to enhanced D band intensity, G and 2D band shifts, and the emergence of new vibrational modes, confirming successful molecule attachment and increasing surface disorder.

Supplementary Information S3.

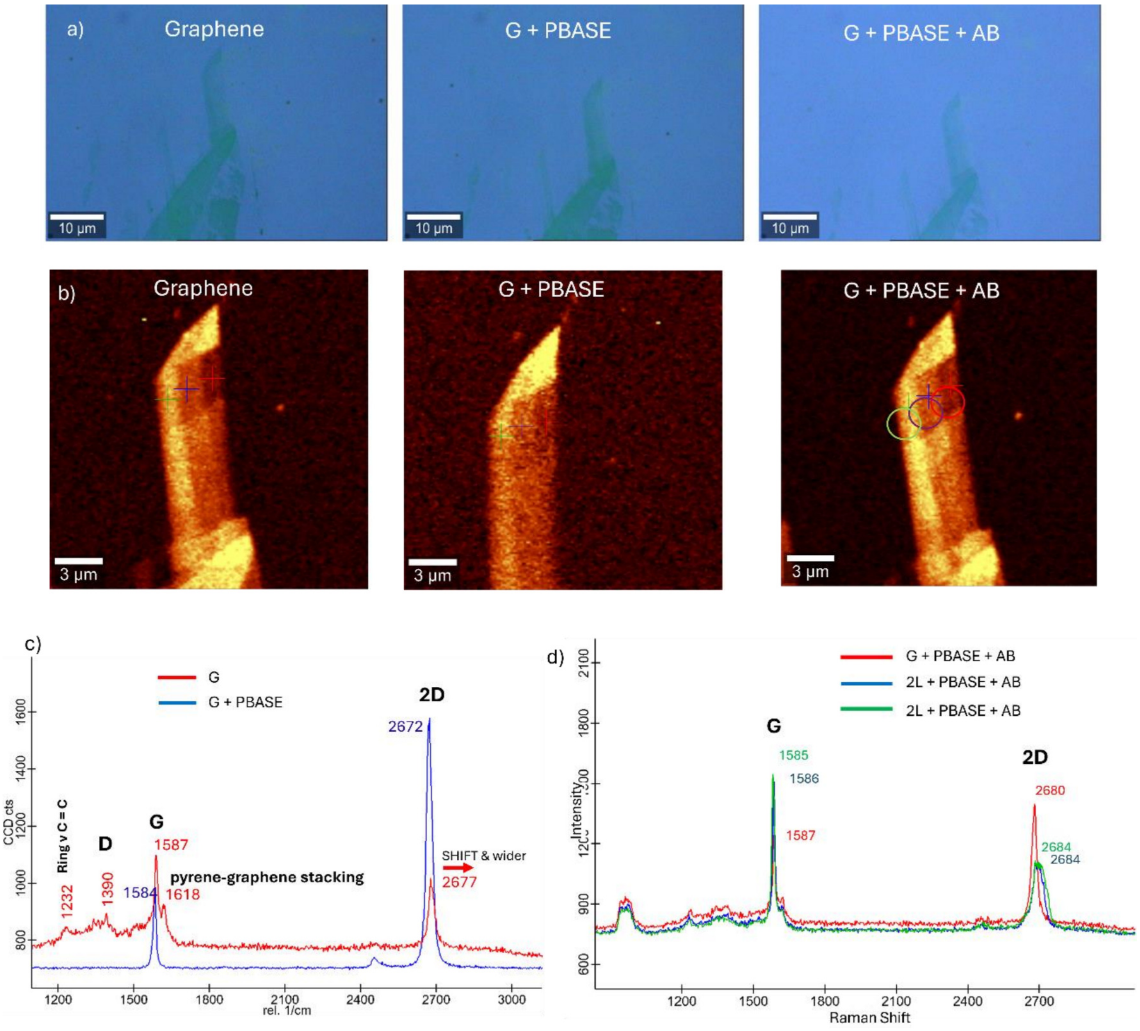

Figure S3. Characterization of graphene functionalization through optical microscopy and Raman spectroscopy. *(a)* Optical microscope images of a graphene flake at three different stages: pristine (Graphene), after functionalization with 1-pyrenebutyric acid N-hydroxysuccinimide ester (G + PBASE), and after antibody immobilization (G + PBASE + AB), showing negligible changes in optical contrast. *(b)* Raman intensity maps of the same flake showing increasing signal upon functionalization, particularly in the G band region, consistent with molecular attachment. *(c)* Raman spectra comparing pristine graphene (red) and G + PBASE (blue), showing the emergence of new peaks at 1232 cm^{-1} and 1618 cm^{-1} (assigned to pyrene) and shifts in the G (1584 → 1587 cm^{-1}) and 2D (2672 → 2677 cm^{-1}) bands, indicating π–π stacking and molecular adsorption. *(d)* Raman spectra comparing monolayer and bilayer graphene regions after functionalization with PBASE and antibody (G + PBASE + AB and 2L + PBASE + AB). Both G and 2D bands show subtle shifts and broadening (e.g., 2D band at 2680–2684 cm^{-1}), confirming consistent surface modification across different graphene thicknesses.

Supplementary Information S4. Raman Intensity enhancement factor E.

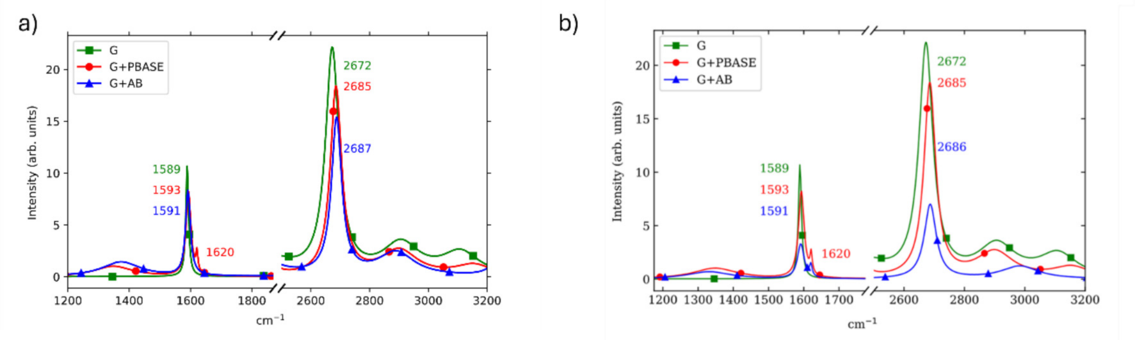

Figure S4. a) Raman spectra of graphene (G), graphene functionalized with PBASE (G + PBASE), and graphene after antibody immobilization (G + AB, where AB corresponds to the SARS-CoV-2 Spike S1 antibody). The spectra show the characteristic G (~1580 cm^{-1}), D (~1620 cm^{-1}), and 2D (~2680 cm^{-1}) bands of graphene on top of one single nanopillar. b) Raman spectra of graphene (G), graphene functionalized with PBASE (G + PBASE), and graphene after antibody immobilization (G + AB, where AB corresponds to the prolactin antibody). The spectra display the characteristic G (~1580 cm^{-1}), D (~1620 cm^{-1}), and 2D (~2680 cm^{-1}) bands of graphene on top of one single nanopillar.

We define the **experimental enhancement factor** as the ratio of the Raman signal measured **on** the nanopillars to that **out** the array under **identical** acquisition conditions (laser power, objective, integration time, grating, focus):

$$E = \frac{\langle S_{\text{on}} \rangle}{\langle S_{\text{out}} \rangle}, S \in \{\text{Peak intensity after baseline subtraction}\}.$$

Because all maps were acquired with the same settings (2 mW, 0.1 s per pixel), no additional normalization by power or time is required.

- **Silicon (521 cm^{-1}) extracted from Fig. 2e:**

  $$E_{\text{Si},521} \approx 3.5\ \times$$

- **Final stacks (after PBASE + antibody) extracted from Fig S4:**
  - **SARS-CoV-2 Spike S1 (G+AB):**

    $E_{2\text{D}} \approx 25\times, E_{\text{G}} \approx 18\times.$
  - **Prolactin (G+AB):**
  - $E_{2\text{D}} \approx 22\times, E_{\text{G}} \approx 16\times.$
